# Association between High Blood Pressure in the Emergency Department and Cryptogenic Hemoptysis

**DOI:** 10.3390/jcm11185302

**Published:** 2022-09-08

**Authors:** Ji Eun Park, Jin A Seo, Jung Guen Cha, Jae Kwang Lim, Jongmin Park, Yong Hoon Lee, Sun Ha Choi, Hyewon Seo, Seung Soo Yoo, Shin Yup Lee, Seung Ick Cha, Jae Yong Park, Chang Ho Kim, Jaehee Lee

**Affiliations:** 1 Department of Internal Medicine, School of Medicine, Kyungpook National University, Daegu 41944, Korea; 2 Department of Radiology, School of Medicine, Kyungpook National University, Daegu 41944, Korea

**Keywords:** hemoptysis, cryptogenic, hypertension, smoking, etiology

## Abstract

Hemoptysis is a common cause of emergency department (ED) visits. There is little data about the role of systemic hypertension as a cause of hemoptysis. The aim of this study was to evaluate the association between systemic blood pressure and the unknown etiology of hemoptysis. This retrospective study included consecutive patients who visited the ED owing to hemoptysis and underwent a chest computed tomography between January 2011 and June 2021. Details of the initial blood pressure at the ED visit were compared between two groups with identified and unidentified causes of hemoptysis. In total, 1105 adult patients were included. The etiology of hemoptysis was identified in 1042 patients (94.3%) and remained unidentified in 63 patients (5.7%). The percentage of patients with severe hypertension was significantly higher in patients with unidentified causes of hemoptysis than in those with identified causes (35% vs. 11%, *p* < 0.001). In multivariate analysis, age, ever-smoker, and initial systolic blood pressure were significantly associated with hemoptysis of unidentified causes. Although further studies are needed, our findings suggest a possible association between high blood pressure and cryptogenic hemoptysis.

## 1. Introduction

Hemoptysis is the expectoration of blood from the lower respiratory tract and is usually an alarming respiratory symptom requiring further evaluation [1,2]. Hemoptysis is caused by various factors, including acute and chronic bronchitis, pneumonia, tuberculosis, bronchiectasis, lung cancer, and pulmonary embolism [1,3]. Diagnostic approaches include clinical evaluations, laboratory tests, chest radiography, chest computed tomography (CT), and bronchoscopy [4]. However, the etiology of hemoptysis sometimes remains undefined despite thorough evaluation, termed ‘cryptogenic hemoptysis’ [5,6].

The incidence of cryptogenic hemoptysis varies widely from 5% to 34%, depending on the studies [3,6,7,8,9,10]. Cryptogenic hemoptysis was previously known to cause minor bleeding; however, recent studies have also revealed cases of massive or life-threatening bleeding that required bronchial artery embolization (BAE) or surgery for bleeding control [6]. However, the long-term prognosis of cryptogenic hemoptysis is considered good [5]. Although several studies have indicated an association between smoking history and cryptogenic hemoptysis [11,12], the clinical characteristics or etiology of cryptogenic hemoptysis remain obscure.

The lungs have dual blood supply known as pulmonary and systemic circulation [13]. Owing to the relatively high pressure of systemic circulation, bronchial circulation is responsible for approximately 90% of significant hemoptysis cases [13,14]. A few case reports have suggested an association between uncontrolled systemic hypertension and alveolar hemorrhage [15,16,17,18,19]. However, the role of systemic hypertension in the etiology of hemoptysis has not been well evaluated. Therefore, in this study, we aimed to evaluate the association between systemic blood pressure and cryptogenic hemoptysis.

## 2. Materials and Methods

### 2.1. Study Population

This study was conducted at Kyungpook National University Hospital, a tertiary referral hospital in South Korea. We retrospectively reviewed the records of all consecutive patients who visited the emergency department (ED) for hemoptysis between January 2011 and June 2021. Adult patients who underwent chest CT for the diagnostic evaluation of hemoptysis were enrolled in this study. Patients with pseudo-hemoptysis who were found to have upper respiratory tract bleeding or upper gastrointestinal bleeding based on the results of laryngoscopy or endoscopy were excluded.

### 2.2. Study Designs

The etiology of hemoptysis was determined by the patient’s clinical and medication history; available laboratory findings including hemoglobin level, platelet count, prothrombin time, activated partial thromboplastin time, creatinine level, antinuclear antibodies, antineutrophil cytoplasmic antibodies, and anti-glomerular basement membrane antibodies; microbiological examination of sputum; chest CT findings; and bronchoscopic results. Cases with no objective evidence of disease or condition causing hemoptysis at the initial examination and throughout the follow-up period were classified as hemoptysis of unidentified causes. We collected detailed data from the medical records—including age, sex, smoking history, initial blood pressure at the ED visit, number of repeated ED visits owing to hemoptysis, the need of BAE or surgical treatment, and hemoptysis-related death—and compared between two groups with identified and unidentified causes of hemoptysis. Severe hypertension was defined as systolic blood pressure ≥180 mmHg and/or diastolic blood pressure ≥120 mmHg upon arrival at the ED [20]. The study protocols were reviewed and approved by the Institutional Review Board of Kyungpook National University Hospital.

### 2.3. Statistical Analysis

Statistical analyses were performed using IBM SPSS Statistics for Windows, Version 22.0 (IBM Corp., Armonk, NY, USA). Continuous variables were expressed as the mean (standard deviation—SD) or median (interquartile range—IQR), and differences between the groups were analyzed using the *t*-test or Mann–Whitney U test. Categorical variables were expressed as absolute values and percentages and analyzed using the χ2 test or Fisher’s exact test. Multivariate logistic regression analysis was conducted using variables with *p*-value < 0.05 in univariate analysis to identify the factors associated with hemoptysis of unidentified causes.

## 3. Results

### 3.1. Etiology of Hemoptysis

In this study, 1105 adult patients with true hemoptysis and available chest CT data were enrolled from all 1896 patients who visited the ED for hemoptysis during the study period. Among them, the etiology of hemoptysis was identified in 1042 patients (94.3%), but the possible cause of hemoptysis was not identified even after precise evaluation, including a drug history and contrast-enhanced chest CT and/or bronchoscopy, in the remaining 63 patients (5.7%). Bronchoscopy was performed in 235 (23%) and 20 (32%) patients with identified and unidentified causes of hemoptysis, respectively (*p* = 0.093). Bronchiectasis (40.2%) was the most common cause of hemoptysis, followed by active tuberculosis (6.9%), malignancy (6.3%), and pneumonia or lung abscess (6.2%). The other identified causes of hemoptysis are listed in Table 1.

### 3.2. Clinical Characteristics and Outcomes of Patients with Identified and Unidentified Causes of Hemoptysis

The clinical characteristics and outcomes were compared between patients with identified and unidentified causes of hemoptysis. Patients with unknown causes of hemoptysis were significantly younger than those with identified causes of hemoptysis (49 ± 15 years vs. 64 ± 15 years, *p* < 0.001), and male sex was more common (76% vs. 61%, *p* = 0.013) (Table 2). Both groups had similar percentage of patients with underlying hypertension; however, the initial blood pressure at ED arrival was significantly higher in patients with unidentified causes of hemoptysis than in those with identified causes of hemoptysis (systolic blood pressure, 165 ± 27 mmHg vs. 146 ± 26 mmHg, *p* < 0.001; diastolic blood pressure, 96 ± 17 mmHg vs. 83 ± 14 mmHg, *p* < 0.001). In particular, the percentage of patients with severe hypertension was significantly higher among those with unidentified causes of hemoptysis (35% vs. 11%, *p* < 0.001). 

In patients with unidentified causes of hemoptysis, BAE was less required to stop hemoptysis (6% vs. 15%, *p* = 0.053). Additionally, 41 (4%) of 1042 patients with identified causes of hemoptysis underwent surgery for treating the underlying etiology or bleeding control, while no patient in the cryptogenic hemoptysis group required surgery. The total number of repeated ED visits and the percentage of patients who visited the ED more than twice owing to recurrent hemoptysis were significantly higher in patients with identified causes of hemoptysis than in those with unknown causes (1.2 ± 0.7 vs. 1.1 ± 0.2, *p* < 0.001; 14% vs. 5%, *p* = 0.037). No hemoptysis-related deaths were reported in patients with unidentified causes of hemoptysis during the follow-up period.

### 3.3. Factors Associated with Hemoptysis Due to Unidentified Causes and Characteristics of Patients with Initial Severe Hypertension

Univariate and multivariate regression analyses were performed to determine factors associated with hemoptysis due to unidentified causes (Table 3). Univariate analysis revealed that young age, male sex, ever-smoker, initial systolic blood pressure, and initial diastolic blood pressure were associated with hemoptysis due to unidentified causes. In the multivariate analysis, age (odds ratio (OR): 0.94, 95% confidence interval (CI): 0.92–0.95, *p* < 0.001), ever-smoker (OR: 2.13, 95% CI: 1.22–3.75, *p* = 0.008), and initial systolic blood pressure (OR: 1.03, 95% CI: 1.02–1.04, *p* < 0.001) were significantly associated with hemoptysis of unidentified causes.

The clinical and radiological characteristics of 63 patients with hemoptysis of unidentified causes were compared according to the presence or absence of initial severe hypertension (Table 4). A previous medical history of hypertension was more common in the severe hypertension group than in the non-severe hypertension group (12 (55%) vs. 11 (27%), *p* = 0.005). Among the 12 patients with a prior history of hypertension presenting with severe hypertension, only seven (58%) had been taking antihypertensive drugs regularly, while five (42%) did not take medication despite knowing their high blood pressure status. Hypertension was diagnosed first after the development of hemoptysis in the remaining ten patients in the severe hypertension group.

None of the patients in the severe hypertension group experienced BAE or recurrent hemoptysis. Chest CT findings were comparable between the severe hypertension and non-severe hypertension groups among patients with unidentified causes of hemoptysis. 

## 4. Discussion

In this study, the common causes of hemoptysis were bronchiectasis, tuberculosis, and malignancy. Despite comprehensive evaluation, the causes of hemoptysis remained unidentified in 5.7% of patients. Higher systolic blood pressure, young age, and ever-smoking status were independently associated with an unknown etiology of hemoptysis. Conservative treatment led to satisfactory outcomes in patients with cryptogenic hemoptysis, and no hemoptysis-related deaths were reported, suggesting a benign prognosis. 

Compared with that of previous studies, our study had a relatively low percentage of patients with unknown causes of hemoptysis. This is probably because all of the patients included in the current study underwent chest CT, which contributed to the identification of the etiology of hemoptysis in more patients. However, our findings were consistent with those of several previous studies addressing the causal relationship between smoking and cryptogenic hemoptysis [11,12]. Smoking-induced chronic bronchial inflammation and hypervascularization within the bronchial wall have been suggested as possible explanations for bronchial bleeding [12]. 

Although uncontrolled hypertension may be a risk factor for hemoptysis as in bleeding of other organs—such as intracerebral hemorrhage, retinal hemorrhage, or epistaxis—the data regarding the association between hypertension and hemoptysis are sparse. A few studies have suggested a possible relationship between high systemic blood pressure and hemoptysis. A study indicated that systemic hypertension was the sole cause of bleeding in two out of 208 patients with hemoptysis, and bleeding was closely related to the fluctuation of blood pressure [2]. In another study of cystic fibrosis patients, beta-blockers significantly reduced the incidence of recurrent hemoptysis, although systemic blood pressure did not significantly decrease after drug administration [21]. The study assumed that the decrease in bronchial artery pressure may have preceded the measurable systemic artery pressure, which prevented recurrent hemoptysis in cystic fibrosis patients who generally have a high propensity toward hemorrhage due to chronic airway inflammation [21,22]. 

It may be argued that high blood pressure is a physiological response to hemoptysis, followed by anxiety-induced adrenergic activation. Similarly, incident severe hypertension is common during admission unrelated to hypertension [23,24]. The proportion of patients with severe hypertension in our hemoptysis cohort (12%) was comparable to that previously reported in a study revealing the prevalence of severe hypertension during the hospitalization of patients admitted for reasons other than hypertension [24]. However, the frequency of severe hypertension in the group with hemoptysis of unidentified causes was three times higher than that in the group with identified causes (35% vs. 11%, *p* < 0.001). Additionally, the initial systolic blood pressure was independently associated with unidentified causes of hemoptysis. Therefore, it can be deduced that uncontrolled hypertension might have contributed to the development of hemoptysis in patients with unidentified causes of hemoptysis.

Diffuse alveolar hemorrhage (DAH) with hemoptysis caused by systemic hypertension or hypertensive emergency has been discussed in several studies [16,17,18,19]. A case report of a young adult who presented with hemoptysis due to a hypertensive emergency suggested a direct bronchopulmonary artery injury from systemic hypertension as one of the several possible mechanisms of DAH [17]. The bronchial arteries terminate at the level of the respiratory bronchioles and communicate distally with the pulmonary capillaries [25,26]. Thus, high systemic blood pressure could contribute to the rupture of the distal microvascular network of the bronchial artery and manifest as alveolar hemorrhage. Almost all reported cases of severe hypertension-associated DAH were in young or middle-aged males [16,17,18,19]. The diagnostic rate of hypertension tends to be lower in young adults, and treatment is often delayed [27]. In this study, over 40% of the patients with unknown causes of hemoptysis presenting with severe hypertension were first diagnosed with hypertension. In addition, a recent study on age-stratified associations between sex and uncontrolled hypertension revealed that men had higher odds of uncontrolled hypertension from age 20–49 years [28]. Young male patients who are more reluctant to take medications are likely to have a higher risk of alveolar hemorrhage caused by uncontrolled severe hypertension. 

Despite the broad clinical spectrum of cryptogenic hemoptysis, from benign to life-threatening, clinical outcomes have been reported to be favorable [5,29]. Similarly, fewer BAE were performed, no surgical treatment was needed for bleeding control, and no hemoptysis-related death was found in patients with unidentified causes of hemoptysis, providing further evidence for the benign nature of cryptogenic hemoptysis. 

The current study has several limitations. First, although only patients with available chest CT data were included in this study, not all patients with hemoptysis underwent bronchoscopy. Therefore, some patients may have been misclassified under the cryptogenic hemoptysis group. However, considering the minimal role of bronchoscopy in patients with hemoptysis who have unremarkable chest CT findings and a benign clinical course, it may not have a significant impact [30]. Second, the flow of diagnostic workup was not controlled and inevitable biases were introduced due to the retrospective nature of the study. Future prospective studies need to be conducted with a standardized diagnostic workup. Moreover, the influence of the blood pressure trends on the course of bleeding in each patient remained unevaluated. Therefore, this study did not provide a direct causality or the mechanism of high systemic blood pressure in the development of bleeding from the respiratory tract. 

## 5. Conclusions

Young age, smoking, and high systolic blood pressure were associated with an unknown etiology of hemoptysis. Although further studies are needed, our findings suggest a possible association between high blood pressure and cryptogenic hemoptysis.

## Figures and Tables

**Table 1:** Etiology of hemoptysis.

Cause	No. of Patients (%)
Identified causes	1042 (94.3)
Bronchiectasis	444 (40.2)
Active tuberculosis	76 (6.9)
Malignancy	70 (6.3)
Pneumonia/lung abscess	69 (6.2)
Bronchial anthracofibrosis	46 (4.2)
Acute bronchitis	30 (2.7)
Aspergilloma	26 (2.4)
Drug-related ^ǂ^	26 (2.4)
Chronic bronchitis	23 (2.1)
Aortic dissection/vascular anomaly	18 (1.6)
Pulmonary hypertension/cardiac	17 (1.5)
NTM lung disease	15 (1.4)
Pulmonary thromboembolism	6 (0.5)
Vasculitis	5 (0.5)
Radiation pneumonia/fibrosis	5 (0.5)
Broncholith	5 (0.5)
Trauma-related	4 (0.4)
Bleeding disorder	4 (0.4)
Catamenial	3 (0.3)
Benign endobronchial tumor	1 (0.1)
Mixed *	149 (13.5)
Without any identified causes	63 (5.7)

Data are expressed as number (%). NTM, non-tuberculous mycobacteria. ^ǂ^ Including single or dual antiplatelet therapy (*n* = 14), oral anticoagulants therapy (*n* = 8), combined antiplatelet agent and anticoagulant therapy (*n* = 3), and heparin (*n* = 1). * Coexistence of two or more of the known causes listed above.

**Table 2:** Comparison of clinical characteristics and outcomes between patients with identified and unidentified causes of hemoptysis.

Variables	Total (*n* = 1105)	Identified Causes (*n* = 1042)	Unidentified Causes (*n* = 63)	*p*-Value
Age, years	64 ± 15	64 ± 15	49 ± 15	<0.001
Male	678 (61)	630 (61)	48 (76)	0.013
Ever-smoker	537 (49)	497 (48)	40 (63)	0.005
Previous HTN	388/1099 (35)	365/1038 (35)	23/61 (38)	0.687
Initial BP				
SBP, mmHg	147 ± 27	146 ± 26	165 ± 27	<0.001
DBP, mmHg	84 ± 15	83 ± 14	96 ± 17	<0.001
SBP ≥ 180 mmHg	126 (11)	104 (10)	22 (35)	<0.001
DBP ≥ 120 mmHg	22 (2)	16 (2)	6 (10)	0.001
Severe HTN *	132 (12)	110 (11)	22 (35)	<0.001
BAE	163 (15)	159 (15)	4 (6)	0.053
Surgery	41 (4)	41 (4)	0 (0)	0.165
Lung cancer	15 (37)	15 (37)	-	
Bronchiectasis	11 (27)	11 (27)	-	
Fungus ball	10 (24)	10 (24)	-	
Actinomycosis	2 (5)	2 (5)	-	
Anthracofibrosis	2 (5)	2 (5)	-	
Necrotizing pneumonia	1 (2)	1 (2)	-	
Recurrent hemoptysis				
No. of repeated ED visits	1.2 ± 0.7	1.2 ± 0.7	1.1 ± 0.2	<0.001
No. of ED visits ≥ 2	149 (14)	146 (14)	3 (5)	0.037
Hemoptysis-related death	38 (3)	38 (4)	0 (0)	0.163

Data are expressed as mean ± standard deviation or number (%). HTN, hypertension; BP, blood pressure; SBP, systolic blood pressure; DBP, diastolic blood pressure; BAE, bronchial artery embolization; ED, emergency department. * SBP ≥ 180 mmHg and/or DBP ≥ 120 mmHg.

**Table 3:** Multivariate analysis of factors associated with hemoptysis of unidentified causes.

	Univariate		Multivariate	
Variable	Odds Ratio (95% CI)	*p*-Value	Odds Ratio (95% CI)	*p*-Value
Age, years	0.94 (0.93–0.96)	<0.001	0.94 (0.92–0.95)	<0.001
Male	2.09 (1.16–3.79)	0.015	—	
Ever-smoker	1.91 (1.13–3.23)	0.016	2.13 (1.22–3.75)	0.008
Initial SBP, mmHg	1.03 (1.02–1.04)	<0.001	1.03 (1.02–1.04)	<0.001
Initial DBP, mmHg	1.06 (1.04–1.07)	<0.001	—	

CI, confidence interval; SBP, systolic blood pressure; DBP, diastolic blood pressure.

**Table 4:** Clinical and radiologic characteristics of patients with hemoptysis of unidentified causes according to the presence or absence of severe hypertension.

Variables	Severe HTN (*n* = 22)	Non-Severe HTN (*n* = 41)	*p*-Value
Age, years	42 (40–61)	46 (37–61)	0.817
Male	14 (64)	34 (83)	0.087
Ever-smoker	12 (55)	28 (68)	0.411
Previous HTN	12 (55)	11 (27)	0.005
Initial BP			
SBP, mmHg	195 (183–205)	145 (140–160)	<0.001
DBP, mmHg	105 (100–120)	93 (80–100)	<0.001
No. of ED visits ≥ 2	0 (0)	3 (7)	0.546
BAE	0 (0)	4 (10)	0.288
Chest CT findings			
Bronchial artery hypertrophy	1 (5)	5 (12)	0.656
Ground-glass opacity	19 (86)	30 (73)	0.343
Consolidation	1 (5)	7 (17)	0.243
Diffuse hemorrhage	2 (9)	2 (5)	0.599
Bilateral involvement	4 (19)	9 (22)	1.0

Data are expressed as median (interquartile range) or number (%). HTN, hypertension; BP, blood pressure; SBP, systolic blood pressure; DBP, diastolic blood pressure; ED, emergency department; BAE, bronchial artery embolization; CT, computed tomography.

## Data Availability

The datasets used and/or analyzed during the present study are available from the corresponding author on reasonable request.
